# Advances and challenges in the field of plasma polymer nanoparticles

**DOI:** 10.3762/bjnano.8.200

**Published:** 2017-09-25

**Authors:** Andrei Choukourov, Pavel Pleskunov, Daniil Nikitin, Valerii Titov, Artem Shelemin, Mykhailo Vaidulych, Anna Kuzminova, Pavel Solař, Jan Hanuš, Jaroslav Kousal, Ondřej Kylián, Danka Slavínská, Hynek Biederman

**Affiliations:** 1 Department of Macromolecular Physics, Faculty of Mathematics and Physics, Charles University, V Holešovičkách 2, 180 00 Prague, Czech Republic; 2 G. A. Krestov Institute of Solution Chemistry of the Russian Academy of Sciences, Akademicheskaya 1, 153045 Ivanovo, Russia

**Keywords:** gas aggregation cluster source, nanocomposite, nanoparticles, plasma polymer, sputtering

## Abstract

This contribution reviews plasma polymer nanoparticles produced by gas aggregation cluster sources either via plasma polymerization of volatile monomers or via radio frequency (RF) magnetron sputtering of conventional polymers. The formation of hydrocarbon, fluorocarbon, silicon- and nitrogen-containing plasma polymer nanoparticles as well as core@shell nanoparticles based on plasma polymers is discussed with a focus on the development of novel nanostructured surfaces.

## Review

### Historical background

“A macromolecule is a molecule of high relative molecular mass, the structure of which essentially comprises the multiple repetition of units derived, actually or conceptually, from molecules of low relative molecular mass.” “A polymer is a substance composed of macromolecules.” These are the definitions the IUPAC gives to macromolecules and polymers, respectively [1]. The ubiquity of polymers in everyday life is due to the huge diversity of chemical composition and architecture they may possess, both factors leading to an extremely broad spectrum of polymer properties. Nevertheless, the word “plasma” was added to the title of this article to reflect the fact that this manuscript will not deal with conventional polymers per se, regardless of the attractiveness and utility of these may be, but will rather focus on materials that are created as a result of a low-temperature non-equilibrium plasma operating in organic vapours. The term “plasma polymer” was introduced in the 1960s to convey the interrelation between the use of a gas discharge and the formation of solid deposits from low molar mass organic precursors [2–4], although the history of plasma polymers is much longer. Organic deposits created as by-products of electrical discharges have been known presumably since the end of the18th century (see [5] and references therein). The significant scientific interest in plasma polymers was motivated by the attractive possibility to introduce various organic monomers into the plasma, including those which do not polymerize by conventional chemical routes. A new kind of polymer with advanced properties was anticipated. It was however soon realized that these materials have little in common with conventional polymers due mainly to the fact that they typically have random, highly cross-linked and highly branched structures in which regularly repeating monomeric units can hardly be expected. The lack of predictable structure hampered the extensive use of plasma polymers in real world applications, although a multitude of potential utilizations have been suggested.

In the mid-twentieth century, such deposits were studied as possible candidates for the production of thin dielectric films for microelectronics [6]. The choice of hydrocarbon, halocarbon and organosilicon precursors in these studies logically stemmed from the requirement of the compatibility with technological processes used in the semiconductor industry. The formation of disperse polymeric particulates in the gas volume was also observed at about that time [7–10]. The phenomenon was earlier considered as unwanted and as something to be avoided; later, it laid a foundation for the field of dusty plasmas in which the research was focused on particle–plasma interactions [11]. A legacy from the semiconductor processing phase explains the fact that close attention was paid to silane-based plasmas [9,11–22] followed by hydrocarbon [16–17,23–27] and fluorocarbon plasmas [27–36].

### Gas aggregation sources

In recent years, scientific interest spread to the investigation of the properties of plasma polymer particles themselves, regardless of the effects their presence produces on the plasma. It was recognized that polymeric nanoparticles (NPs) can be highly desired in various fields including photonics [37] and biomedical applications where they can be used as biomolecule and drug carriers [38–40]. Gas aggregation cluster sources (GAS) were considered feasible for the synthesis of plasma polymer NPs with a tuneable size distribution, retention of functional groups and cross-link density. The concept of GAS was originally developed for the production of metal NPs by vacuum thermal evaporation with subsequent condensation of atomic metal vapours on a cool buffer gas and later thermal evaporation was replaced by magnetron sputtering [41]. At least one work investigated the formation of polymeric NPs by thermal evaporation of poly(*N*-vinyl-2-pyrrolidone) in a GAS [42].

**Figure 1:**
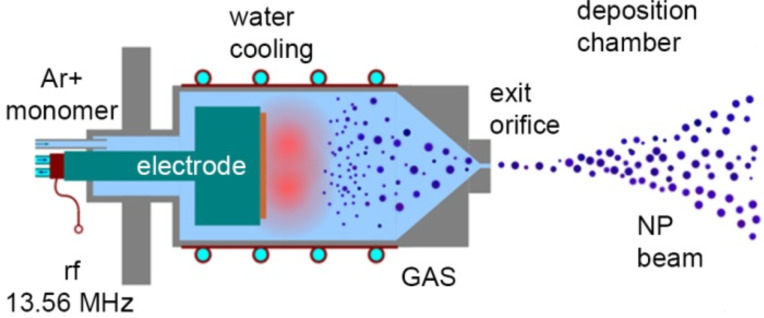
Scheme of a gas aggregation cluster source.

At present, a typical GAS consists of a tubular vacuum chamber equipped with a DC or RF electrode (or magnetron) which is used to ignite a plasma and which serves as a source of material to be “vaporized” (Figure 1). In the case of the production of plasma polymer NPs, carbonaceous precursors are introduced into the GAS in the form of volatile vapours of organic monomers or as a result of evaporation or sputtering of a solid polymer target attached to the electrode. The latter process was shown to lead to a release of volatile fragments of macromolecules which can be further used as precursors for plasma polymerization [43–52]. Depending on the experimental conditions, plasma polymerization can be forced to proceed in a gas phase which results in the formation of NPs of different chemical and physical properties and with different size distribution. The GAS configuration offers an advantage of creating a co-axial gas flow to transport the NPs away from the discharge zone through an orifice into another vacuum chamber where they can be collected on solid supports.

**Figure 2:**
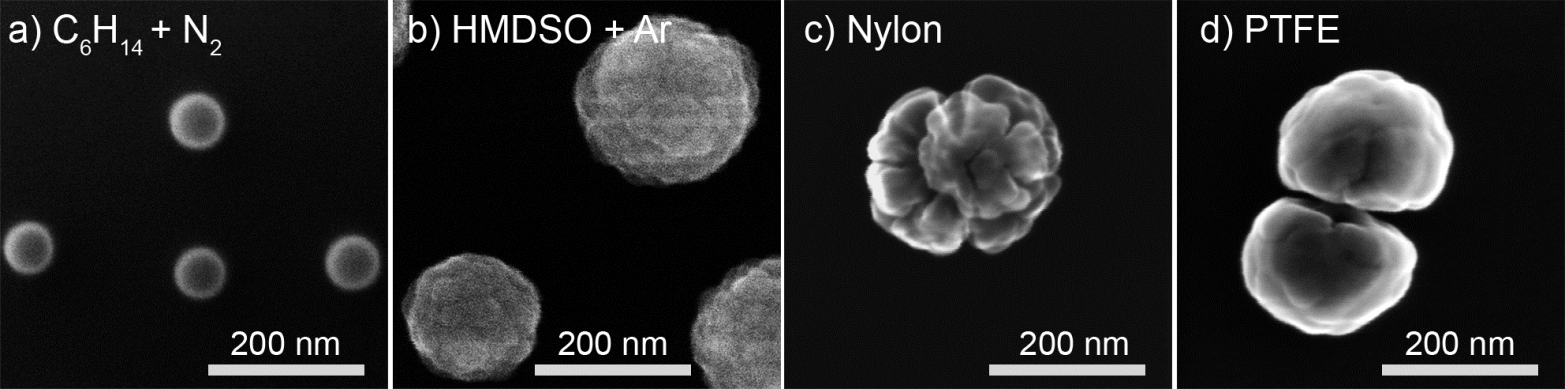
SEM images of different types of plasma polymer nanoparticles produced: a) by plasma polymerization of *n*-hexane in its mixture with N_2_; b) by plasma polymerization of HMDSO in its mixture with Ar (obtained in a similar manner as [53]); c) by RF magnetron sputtering of nylon in the Ar/N_2_ mixture (republished from [54] with permission IOP Publishing Ltd.); d) by RF magnetron sputtering of PTFE in Ar (obtained in a similar manner as [55]). The references shown here and in the following figures cite the authors’ previous works where similar (but not necessarily identical) data were presented; the unreferenced data represent the authors’ new material which has not been published yet, but which is necessary for a comparative analysis in this review.

Figure 2a–d shows scanning electron microscopy (SEM) examples of NPs created as a result of plasma polymerization of *n*-hexane and hexamethyldisiloxane (HMDSO) [53] or as a result of RF magnetron sputtering of nylon [54] and poly(tetrafluoroethylene) (PTFE) [55]. One can readily judge the diversity of shape and morphology of the NPs with diameters ranging from tens to hundreds of nanometers. Here and further in this Review, for simplicity, we shall use the designation “NPs” to describe all particles in this size range having in mind that objects of hundreds of nanometers are more accurately described as submicrometer-sized particles.

### Charge of plasma polymer nanoparticles

It has been recognized that NPs grow via a three-step process involving nucleation, coagulation and growth by accretion. The nucleation stage is often considered to be governed by negative ions [19,56–59], leading to the formation of nanometer-sized nuclei. These embryonic clusters may be neutral or bear either negative or positive charge [60–61] which results in their effective coagulation into larger NPs with a typical diameter of 10–20 nm. Negative charge begins to dominate for NPs of this and larger size because of the high mobility of electrons with respect to positive ions. The coagulation is therefore suppressed by Coulomb repulsion, and further NP growth proceeds by accretion via the accumulation of polymer-forming neutral species (radicals) and positive ions from the gas phase.

**Figure 3:**
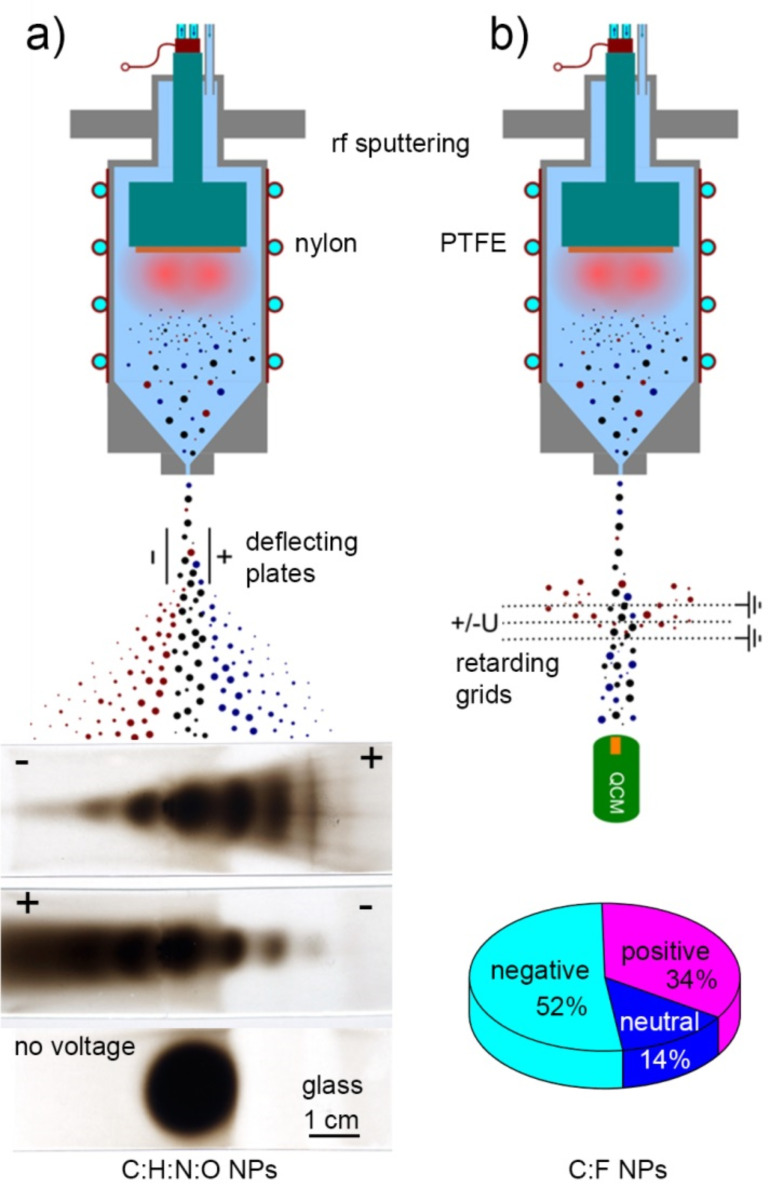
Experimental arrangements allowing estimation of plasma polymer NP charging: a) electrostatic plates used for deflection of charged nylon-sputtered NPs [66] by applying 200 V voltage of different polarity across the NP beam; b) electrostatic grids used for retardation of charged PTFE-sputtered NPs by applying 1000 V voltage of different polarity along the NP beam (obtained in a similar manner as [55]).

The resultant plasma polymer NPs have a spherical symmetry but can exhibit different morphology. Although it is very difficult to generalize about the shape of the NPs prepared in different experiments, it seems that larger plasma polymer particles typically reveal a more complex structure (Figure 2), the exact understanding of which is still lacking. The phenomenon can be associated with the changes in the heat balance of NPs during their growth in plasma. It has been shown both theoretically and experimentally that smaller NPs may reach the temperature that significantly exceeds that of a neutral gas, whereas larger NPs are heated much less [58,62–63]. Thus, the continuous growth of NPs in plasma may be accompanied by radially directed changes in the material properties (cross-link density and branching) induced by temperature changes and resulting in the accumulation of mechanical stress. If the critical value of stress is achieved, the surface of a NP relaxes with the formation of the surface instabilities, similar to a popcorn effect observed in conventional polymer particles [64]. As it was mentioned, the involvement of low-temperature plasma represents a unique feature that distinguishes this approach from other non-plasma-based methods: NPs acquire an electrical charge when nucleating, growing and passing through the zone of the glow discharge. Clouds of charged NPs may exhibit collective behaviour coupled with plasma instabilities, a phenomenon of high scientific interest in the field of dusty plasmas [52]. In the framework of GAS, the gas flow conditions are deliberately chosen to overcome trapping of NPs by electromagnetic fields and to extrude beams of NPs into the separate deposition chamber. Haberland [65] was one of the first to realize that NPs (metallic in his case) ionized in the magnetron plasma can be advantageous in terms of size separation in accord with their mass-to-charge ratio. It can be shown that plasma polymer NPs also leave GAS partially charged and can be manipulated by an electrostatic field. Electrostatic plates can be installed at a close distance from the exit orifice of the GAS to deflect the charged NPs. Figure 3a shows the scheme of such arrangement used for the production of NPs by RF magnetron sputtering of nylon [66]. Here, photos of glass substrates taken after the depositions with or without the electrostatic field applied are shown. The nylon-sputtered NPs produce a circular deposit opposing the exit orifice when no voltage is applied to the deflection plates. In contrast, the deposit becomes spread towards the edges when 200 V voltage of different polarity is applied to the deflection plates. Remarkably, the presence of both negatively and positively charged NPs can be observed as the deposit is smeared in both directions from the central point. From the opacity of the deposit, it can be qualitatively estimated that neutral NPs are in minority and that negatively charged NPs constitute the majority of all the charged NPs.

For a quantitative evaluation, a system of electrostatic retarding grids can be utilized as shown in Figure 3b. Quartz crystal microbalance (QCM) can measure the total mass flux of the NPs (neutral, positively and negatively charged) without any voltage applied to the grids. A highly positive or negative potential applied to the central grid repels the NPs of the opposite charge and allows the rest to pass through. One can obtain the ratio between the neutral, the positively and the negatively charged NPs by measuring their mass fluxes with opposing voltages on the central grid. The calculations performed for the PTFE-sputtered NPs [55] support the qualitative data obtained for nylon sputtering and prove that the majority of the NPs bear the electric charge and that negatively charged NPs are the most abundant.

### Control of chemical composition of plasma polymer nanoparticles

**Figure 4:**
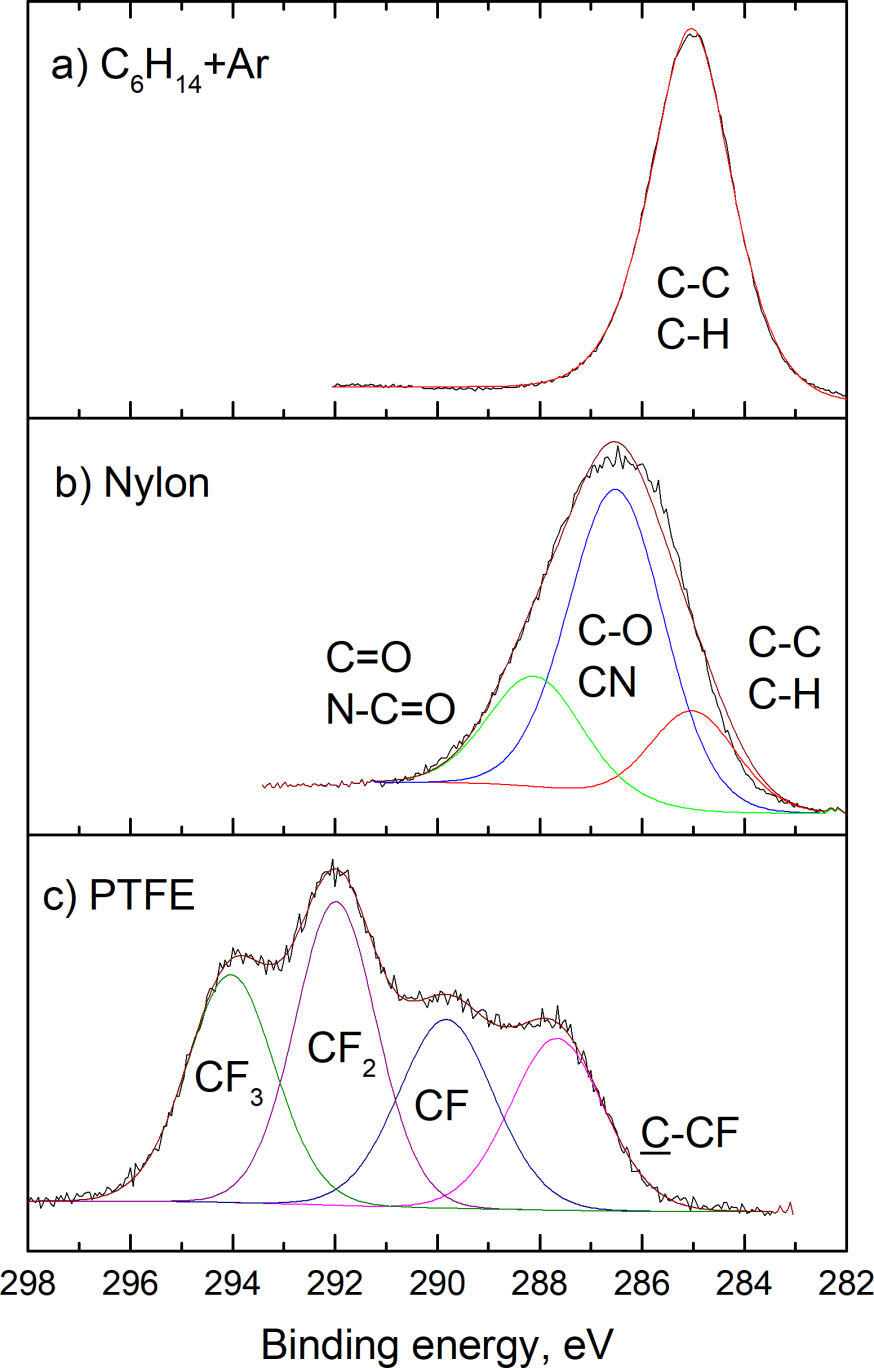
C 1s XPS of the NPs prepared a) by plasma polymerization of *n*-hexane in its mixture with Ar (total pressure 88 Pa, discharge power 40 W, C_6_H_14_ flow 1.2 sccm, Ar flow 12.2 sccm); b) by RF magnetron sputtering of nylon in the Ar/N_2_ 3:1 mixture (obtained in a similar manner as [54]); c) by RF magnetron sputtering of PTFE in Ar (reprinted from [67], with permission from Elsevier).

Chemical composition can also vary drastically depending on the precursor/target used as can be seen in Figure 4 where high-resolution C 1s XPS peaks are shown for a number of chosen NPs. Starting from C/H plasma polymers (Figure 2a), the chemistry of the resulting NPs may range from nitrogen-containing (Figure 2b) to fluorocarbon (Figure 2c) plasma polymers, to cite just a few, in which multitudes of chemical bonding environments can be present.

**Figure 5:**
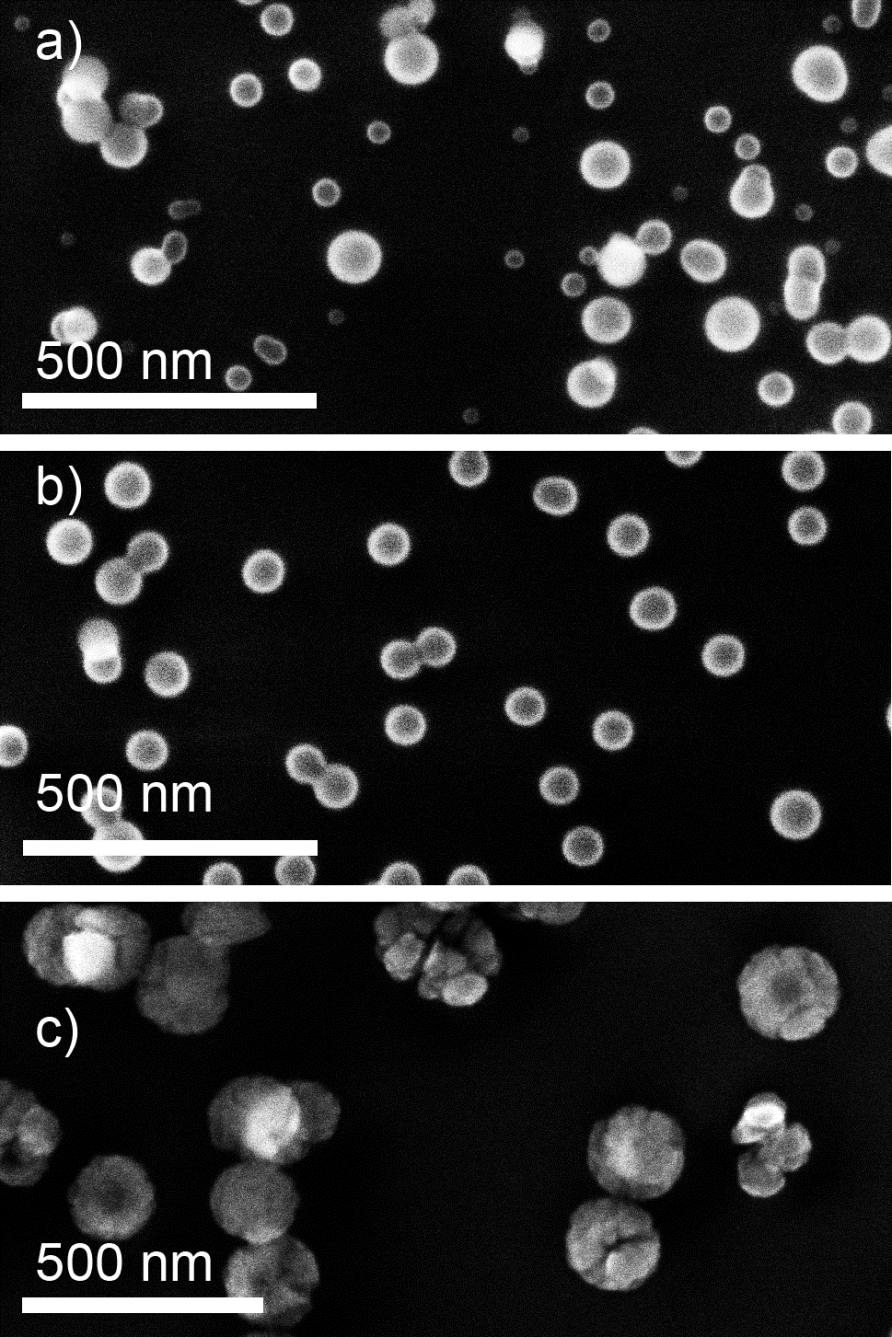
SEM images of nitrogen-containing NPs prepared a) by plasma polymerization of *n*-hexane in its mixture with Ar (total pressure 88 Pa, discharge power 40 W, C_6_H_14_ flow 1.2 sccm, Ar flow 12.2 sccm); b) by plasma polymerization of *n*-hexane in its mixture with N_2_ (total pressure 88 Pa, discharge power 40 W, C_6_H_14_ flow 1.2 sccm, N_2_ flow 12.2 sccm); c) by RF magnetron sputtering of nylon in the Ar/N_2_ 3:1 mixture (obtained in a similar manner as [54]).

The choice of the working gas strongly influences the plasma chemistry and may be used as a tool for tuning the chemical composition of resultant NPs. For example, adding nitrogen to a hydrocarbon plasma may trigger the formation of nitrogen-containing NPs [68–69]. Figure 5a,b shows the NPs produced by plasma polymerization from the mixtures of *n*-hexane with Ar and with nitrogen, and for comparison Figure 5c shows the NPs produced by RF magnetron sputtering of nylon in the Ar/N_2_ mixture [54].

**Figure 6:**
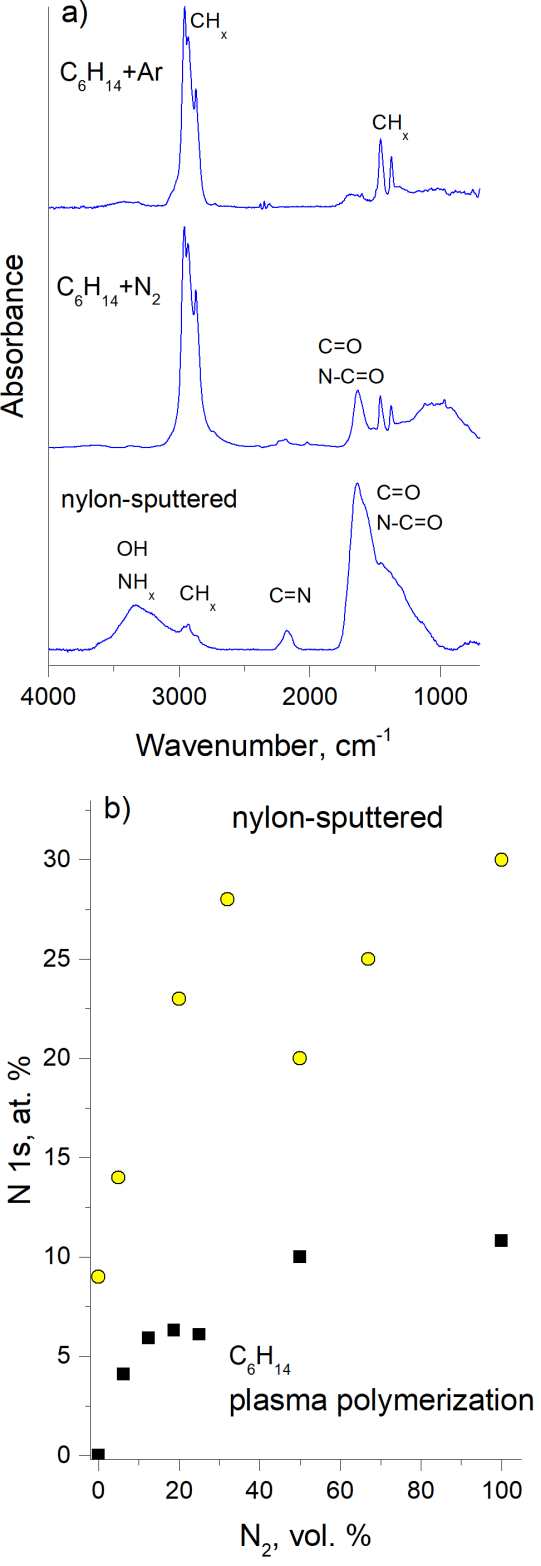
Chemical composition of nitrogen-containing NPs shown in Figure 5: a) FTIR spectra; b) the nitrogen content calculated from the XPS spectra as a function of the N_2_ concentration in the working gas.

The chemical composition of these NPs is shown in Figure 6a in terms of FTIR spectra and in Figure 6b in terms of the XPS elemental nitrogen content as a function of the concentration of N_2_ in the working gas. Apart from narrowing the size distribution, adding nitrogen results in an increase of the nitrogen content in the NPs. Incorporation of nitrogen-bearing species into thin films of plasma polymers has been considered to be of paramount importance, especially in terms of retention of primary amines which are attractive in biomedical applications as linkers for binding biomolecules. Yet, it has been recently argued that primary amines find it difficult to survive the influence of the plasma and that amino groups overwhelmingly reported for plasma polymers are actually other nitrogen-containing functionalities [70]. The data obtained for the NPs confirm that plasma polymerization of *n*-hexane in N_2_ does not lead to the substantial retention of amines as can be seen from the absence of the characteristic IR band at >3000 cm^−1^. Carbonyl-, amide- and, to a lesser extent, imine-based functionalities constitute an ensemble of nitrogen-bearing species. In contrast, RF magnetron sputtering of nylon in the Ar/N_2_ mixture results in a much better retention of nitrogen which is, at least to some extent, bound in amine functionalities. Nevertheless, a reliable and quantitative control over the amount of amines in both NPs and thin films of plasma polymers still represents a formidable challenge.

**Figure 7:**
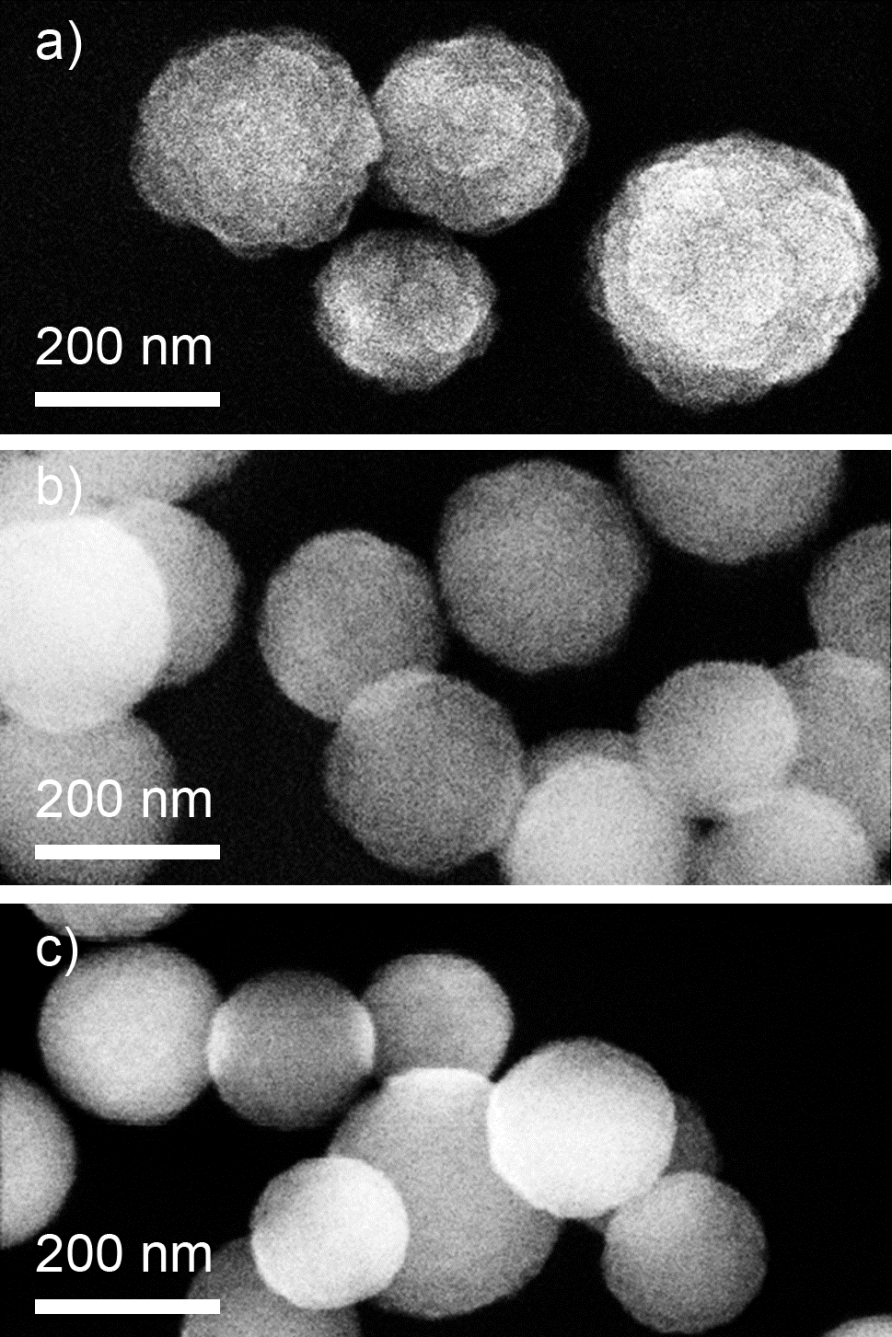
SEM images of NPs prepared by plasma polymerization of HMDSO mixed with Ar: a) without adding oxygen; b) with addition of oxygen at O_2_/HMDSO 1:1 ; c) with addition of oxygen at O_2_/HMDSO 5:1 (obtained in a similar manner as [53]). Total pressure is 55 Pa, discharge power is 30 W, HMDSO flow is 0.2 sccm, Ar flow is 2 sccm.

Another example of a strong dependence of the chemical composition of NPs on the composition of the gas mixture can be found for plasma polymerization of HMDSO. It has been known for a long time in the thin film deposition community that adding oxygen to HMDSO switches plasma chemistry to preferential oxidation of carbonaceous species. A pumping system effectively evacuates gaseous carbon oxides whereas siloxane moieties tend to adsorb on surfaces and form silicon oxide coatings. The amount of added oxygen determines the chemical composition of the coatings. The same paradigm can be adapted for the synthesis of NPs in the configuration of GAS [53]. Plasma polymerization can be performed at elevated pressure in a mixture of HMDSO and Ar with the constant ratio of both components. The process results in the formation of 210 ± 40 nm diameter spherical particles with a more structured surface (Figure 7a). Adding O_2_ to the working mixture results in morphological changes and produces NPs with fewer irregularities on the surface (Figure 7b,c). Remarkably, the NP diameter does not change significantly and reaches 180 ± 40 nm for the most oxygen-rich gas mixture.

**Figure 8:**
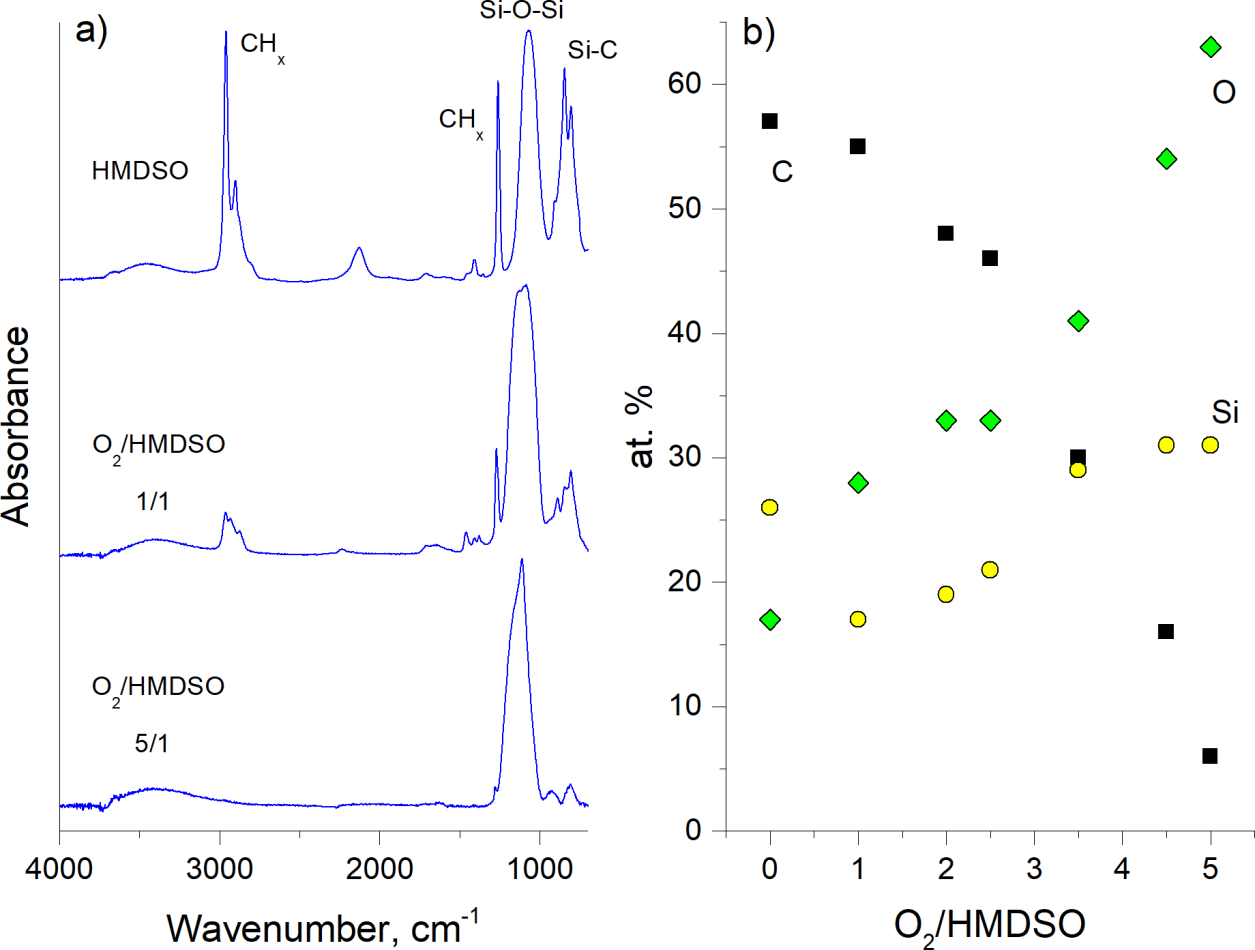
Chemical composition of the NPs shown in Figure 7: a) FTIR spectra b) the elemental content calculated from the XPS spectra as a function of the O_2_ concentration in the working gas (republished from [53] with permission of IOP Publishing Ltd.).

The stability of the size distribution becomes even more remarkable when compared to the chemical changes induced by the addition of oxygen. FTIR and XPS (Figure 8a,b) analyses demonstrate the organosilicon character of the NPs produced without O_2_ and its gradual transfer to the inorganic state with the addition of O_2_. The gas phase composition can be optimized to produce nearly stoichiometric SiO_2_ NPs (Figure 7c), which are rare examples of organic plasma-derived material with well-established chemical composition. As it was discussed above, plasma polymers are typically cross-linked macromolecular networks having the chemical composition of more diverse character than that of the precursors. Mono-functional plasma polymers are still beyond reach and even preferential retention of a specific functional group with a good control over the surface chemistry represents a significant challenge for the scientific community today.

### Control of size distribution of plasma polymer nanoparticles

**Figure 9:**
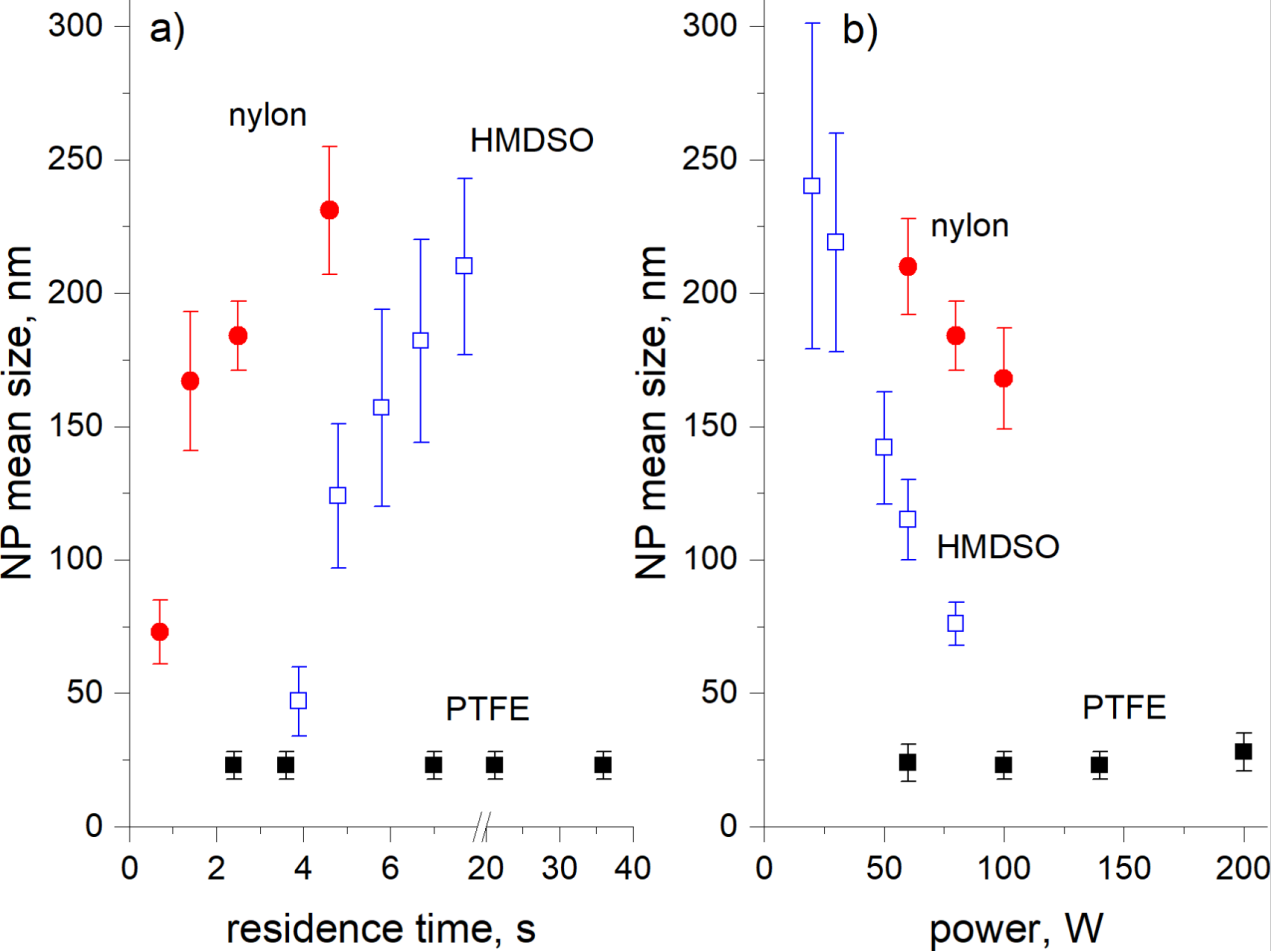
Mean diameter of nylon-sputtered, PTFE-sputtered and HMDSO plasma polymer NPs as a function of a) the residence time and b) the power of the discharge. The data are calculated from the results presented in [53–54,67].

The discharge power and the gas flow in the GAS are additional parameters to control the NP properties. In close analogy with the Yasuda parameter (see [71] and the following debate), both determine the specific energy supplied to a precursor molecule and, as a result, the intensity of precursor fragmentation. A typical pressure of tens of Pa ensures a viscous and laminar gas flow regime, and hence the gas flow rate determines also the time that NPs spend in the GAS (the residence time). Figure 9a,b summarizes the data available in the literature. Here, the mean NP diameter is given as a function of the residence time and discharge power for nylon- [54] and PTFE-sputtered [67] NPs as well as for plasma polymerization of HMDSO in Ar [53].

**Figure 10:**
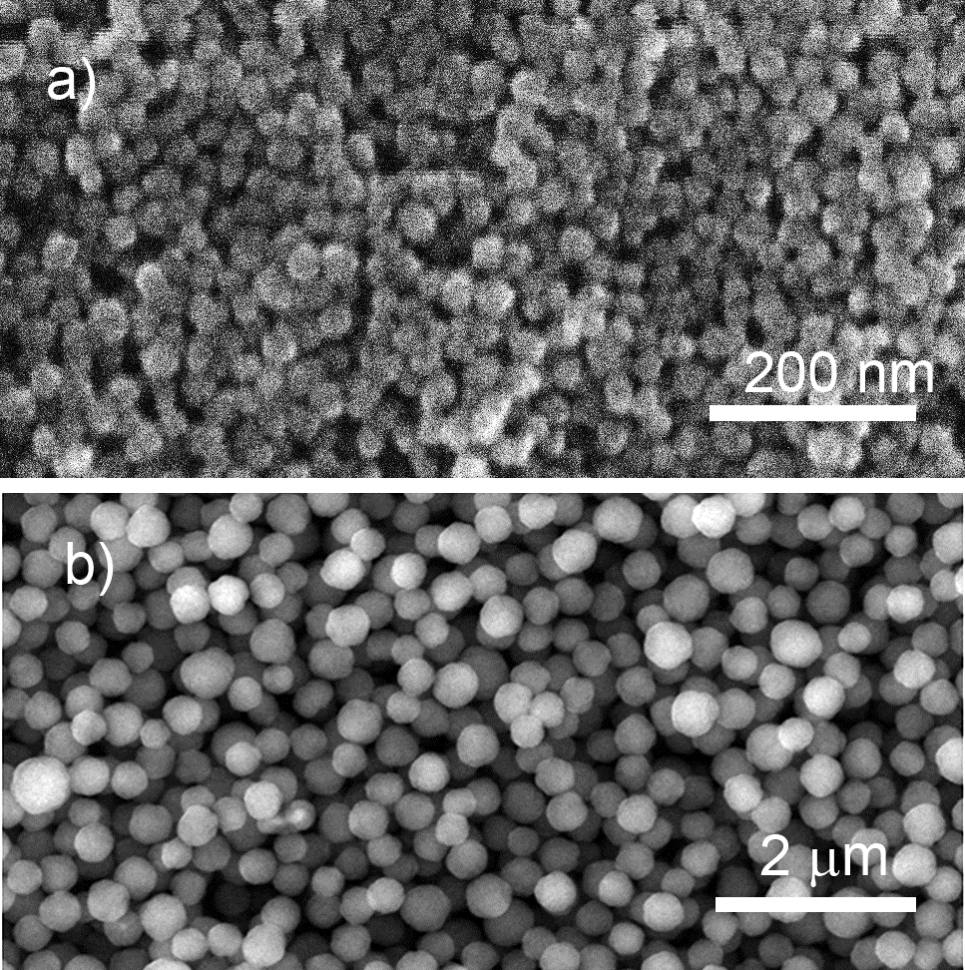
SEM images of the PTFE-sputtered NPs deposited with different intensity of the magnetic field: a) 100 G, b) 250 G; Ar pressure is 100 Pa, flow rate is 9.2 sccm, residence time is 9 s, discharge power is 140 W, deposition time is 20 min; obtained in a similar manner as in [55].

For nylon and HMDSO, an increase is observed of the NP diameter with the residence time (under constant power) which simply reflects the kinetics of the NP growth when they travel along the GAS. The opposite trend of decreasing NP diameter with the discharge power is readily explained by stronger fragmentation of precursor molecules. The fragmentation results in a larger amount of free radicals that serve as nucleation centres and, under constant supply of the precursor (constant gas flow rate), these produce larger amounts of smaller NPs. Nevertheless, the data for the NPs produced by magnetron sputtering of PTFE [67] by no means obey the above trend. These NPs are also in contradiction with other fluorocarbon NPs produced by plasma polymerization of heptadecafluorodecyl acrylate [35] showing the same trend as the nylon-sputtered and the HMDSO NPs. For PTFE-sputtered NPs, neither the residence time nor the discharge power has influence on the NP diameter which stays constant over the entire range of both parameters. The formation of the NPs in close proximity to the PTFE target and their subsequent transport through the GAS volume saturated with low sticking probability CF_2_ bi-radicals were suggested as possible explanations of the phenomenon. It can be concluded that, although the opposing influence of the residence time and the discharge power on the NP diameter is fulfilled in many cases, a global generalization should be made with caution and each particular combination of precursor and GAS parameters should be thoroughly investigated. For example, other experiments with PTFE-sputtered NPs revealed that their diameter can be controlled over a wide range by changing the intensity of the magnetic field above the magnetron target [55]. Figure 10a,b shows SEM images of the NPs prepared under identical conditions in the GAS but with different permanent magnet circuits installed in the magnetron, giving either a 100 G or 250 G field above the position of the erosion track on the PTFE target. An increase of the intensity of the magnetic field leads to a decrease of the magnetron self-bias from 620 V to 350 V due to more effective trapping of electrons within the magnetic channel. This in turn results in the formation of particles which are an order of magnitude larger (250 nm) as compared to the ones fabricated with the weaker magnetic field (30 nm). Apparently, the differences in intensity of ion bombardment should be manifested in the change of the plasma chemistry, although the exact reason for this interesting phenomenon is still not clear and requires further investigation.

**Figure 11:**
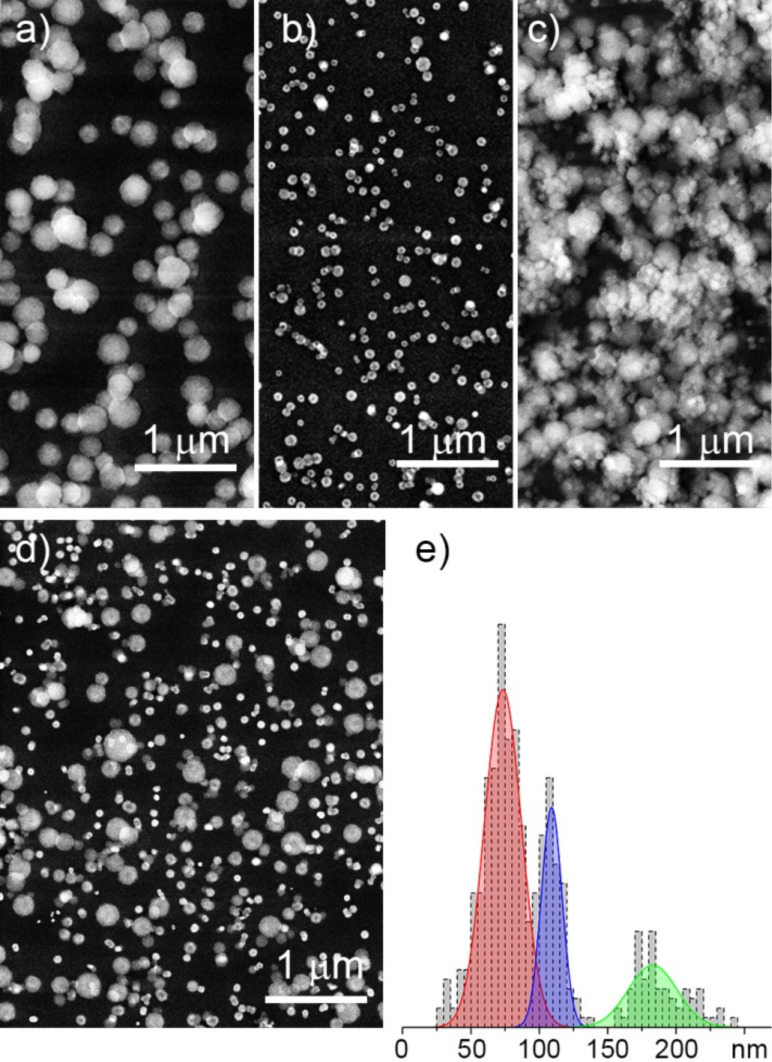
SEM images of NPs prepared by plasma polymerization of HMDSO in Ar: a) 220 nm NPs produced with 10 cm aggregation length; b) 40 nm NPs produced with 4 cm aggregation length; c) dual-scale structure produced by sequential deposition of a) and b); d) triple-scale structure produced by power-dependent sequential deposition of 200, 110 band 70 nm diameter NPs; e) size distribution histogram corresponding to d) (republished from [53] with permission of IOP Publishing Ltd.).

Apart from being of scientific interest, tuning the NP size by replacement of magnetic circuits can hardly be viewed as practical and technological reasons. More feasible is to control the power/flow parameters or to modify the construction of the GAS itself. A GAS can be constructed to allow the length of the aggregation chamber to be changed, and Figure 11a,b shows the results of plasma polymerization of HMDSO in Ar with two values of the aggregation length [53]. Logically, a shorter aggregation zone reduces the NP residence time and prevents them from growing larger. Figure 11c shows the dual-scale surface obtained as a result of the combined deposition when a layer of 220 nm NPs was prepared with the longer aggregation zone and it was subsequently over coated by another layer of 40 nm NPs prepared with the shorter aggregation zone. Manipulation of the discharge power can also be effective for the creation of dual- and even multi-scale structures. For example, NPs of three different sizes can be prepared in a single run by a stepwise increase of power, in this case resulting in the deposition of 200, 110 and 70 nm NPs. Figure 11d shows the outcome of such a triple deposition method and the histogram in Figure 11e confirms the formation of the triple-scale surface. Thus, plasma polymer NPs produced by GAS prove to be very versatile for the design of hierarchical structures, which can be very efficient for fine tuning of optical properties, surface wettability, interaction with cells, and in other applications.

### Core@shell nanoparticles

**Figure 12:**
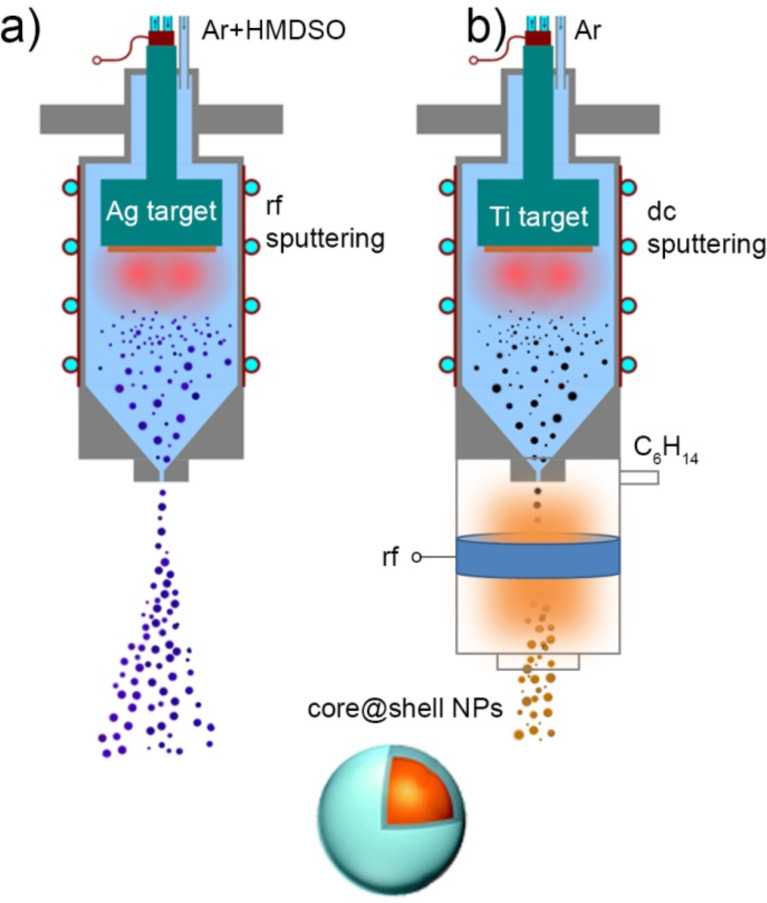
Scheme of synthesis of core@shell NPs by GAS: a) core@shell NPs are produced in the GAS by RF magnetron sputtering of Ag target in the gas mixture of Ar and HMDSO; b) metal NPs are produced by DC magnetron sputtering of Ti target in the GAS and then covered by shells of C/H plasma polymer in the auxiliary plasma reactor with external RF excitation.

The versatility of GAS may be further extended if two different processes are combined in one experimental run. One can take advantage of magnetron sputtering of metals and plasma polymerization of organic precursors to create heterogeneous NPs in which metallic inclusions are enveloped by layers of plasma polymer (core@shell NPs). For example, the process can be performed in the GAS by RF magnetron sputtering of metal in argon with the addition of an organic precursor (Figure 12a) or metal NPs can be pre-formed in the GAS by DC magnetron sputtering and the beam of the NPs can be allowed to pass through an auxiliary glow discharge in organic vapours (Figure 12b).

**Figure 13:**
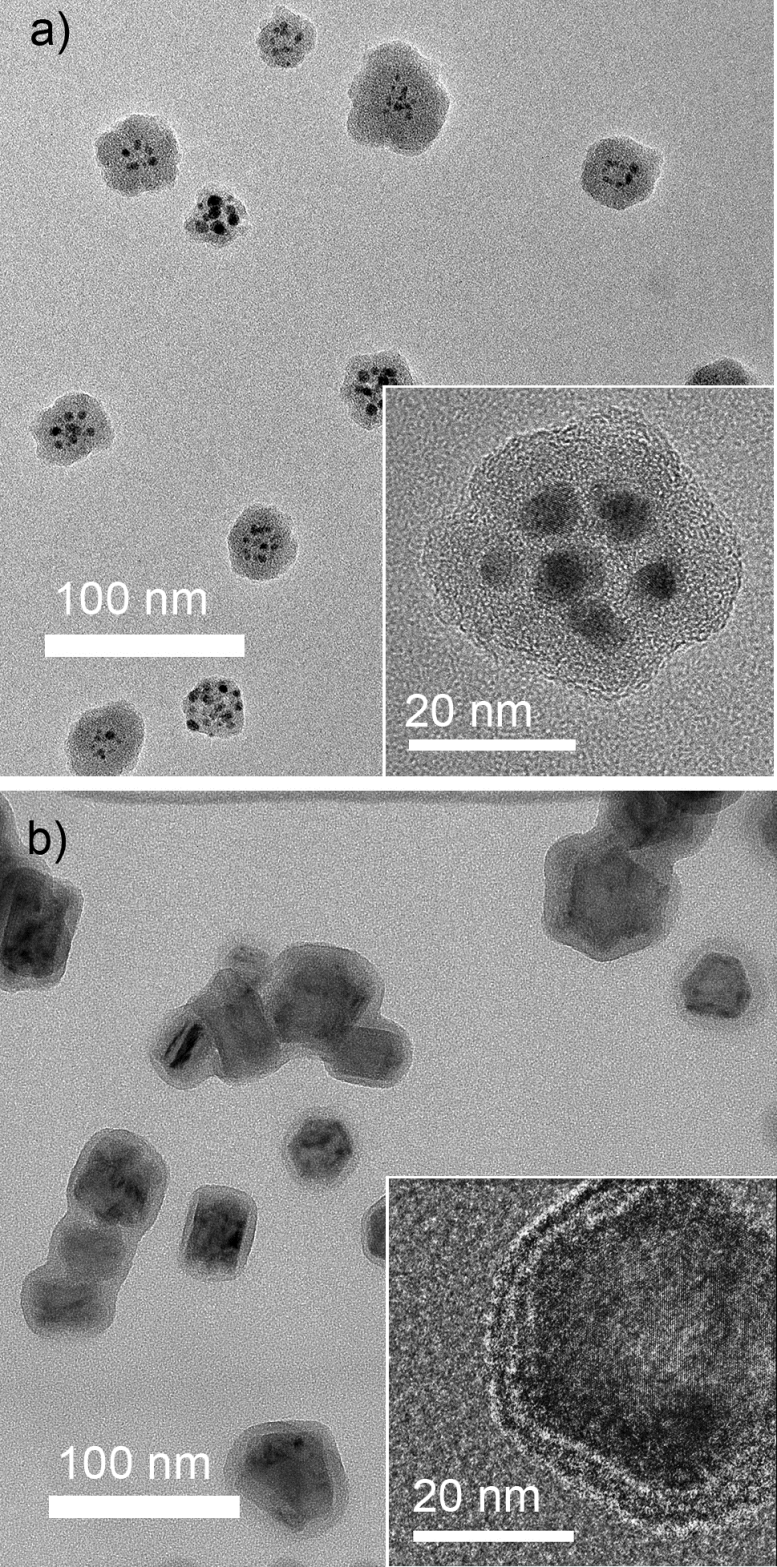
TEM images of core@shell NPs: a) Ag@HMDSO NPs prepared in configuration of Figure 12a (total pressure 190 Pa, discharge power 50 W, HMDSO flow 0.45 sccm, Ar flow 105 sccm); b) Ti@C/H NPs prepared in configuration of Figure 12b (Ar pressure/flow in the GAS is 40 Pa/4.0 sccm, DC 0.4 A, total pressure in the auxiliary plasma zone is 1 Pa (0.65 Pa of Ar and 0.35 Pa of C_6_H_14_), RF power is 10 W).

In the first case, the conditions should be optimized to provide the supply of atomic metal supersaturated vapours into the gas phase where they start to condense by homogeneous nucleation. Simultaneously, fragmentation of organic molecules in the plasma proceeds with the formation of free radicals that subsequently recombine to create the plasma polymer phase. Remarkably, the two processes do not interfere, probably due to strong cohesive forces between metal atoms and weak metal–polymer interaction. As a result, phase-separated core@shell NPs are created, an example of which is shown in Figure 13a. Here, silver NPs enveloped by shells of HMDSO plasma polymer are shown. The structure of the NPs, in which multiple metal inclusions of about 5 nm diameter are concentrated within a single plasma polymer shell producing 36 nm diameter multicore@shell NPs, is appealing. The multicore-in-one-shell structure can be explained by the initial formation of single core@shell NPs which subsequently coalesce into one bigger NP joining multiple metal inclusions in a single shell.

The second strategy relies on spatial separation of the formation of metal NPs and their embedding into polymer or plasma polymer shells, similar to what has been realized in [72–73]. This strategy allows the decoupling of the processes of magnetron sputtering and plasma polymerization and may prove advantageous, especially if more reactive metals are considered. For example, titanium is known to form strong TiC bonds when sputtered in organic plasma [74]. Carbidization of titanium atoms may hinder metal–polymer phase separation and it may even change the properties of titanium inclusions themselves. Instead, Ti NPs can be created in the GAS by magnetron sputtering in Ar and then their beams can be transported by the gas flow through an auxiliary glass tube attached to the GAS and equipped with an external ring electrode for RF excitation of plasma. Vapours of *n*-hexane are introduced into the glass tube via a separate inlet port and the conditions of plasma polymerization should be optimized to produce in-flight coating of Ti NPs with hydrocarbon plasma polymer. Figure 13b shows that both single core@shell NPs as well as their agglomerations can be obtained by this procedure. The thickness of the overcoat can be controlled by the flight velocity of the NPs and by the deposition rate of the plasma polymer. Overall, gas-phase fabrication of metal–polymer core@shell NPs may offer new possibilities in preventing metal particles from oxidation, in fine tuning the optical properties and biological interactions, and, in general, in designing novel materials with advanced properties.

### Plasma polymer nanoparticles as building blocks for nanostructured composite surfaces

**Figure 14:**
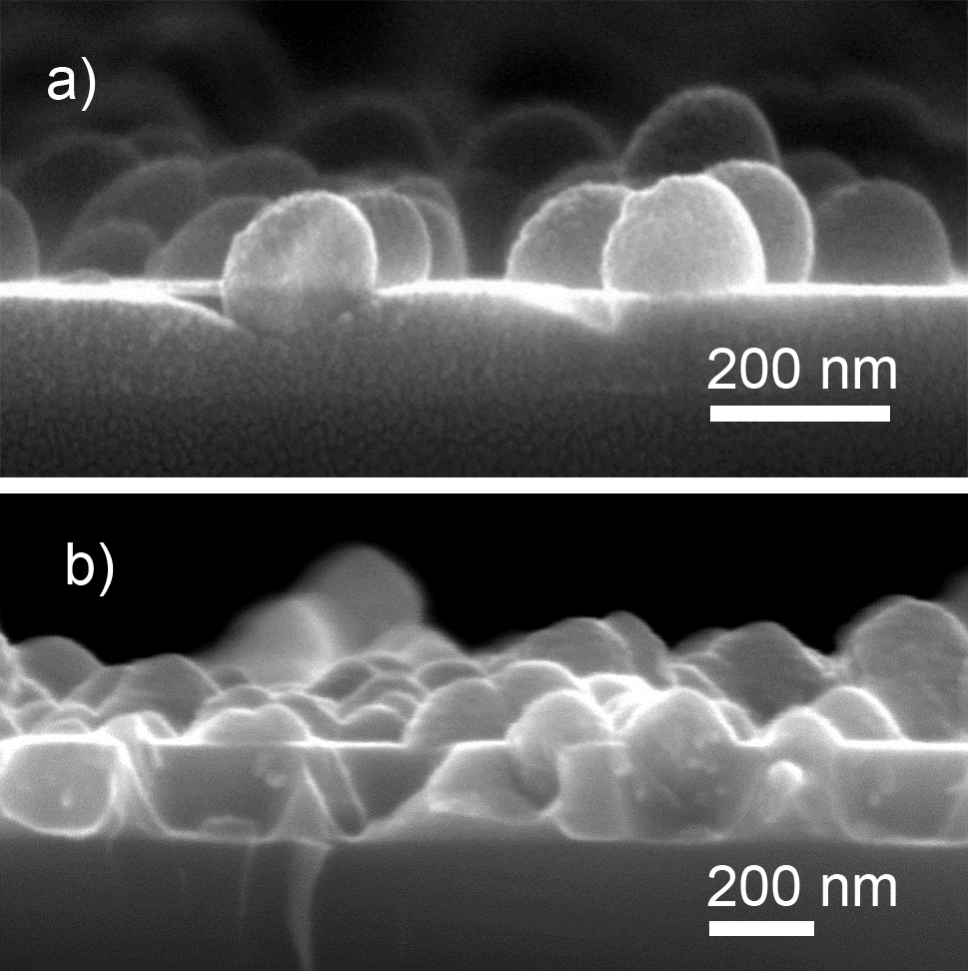
C/H NPs prepared in GAS by plasma polymerization of *n*-hexane and overcoated a) with a thin film of C/H plasma polymer (reprinted from [76], with permission from Elsevier) and b) with a Ti thin film (obtained in a similar manner as in [77]).

The configuration of GAS allows the deposition of NPs onto any high-vacuum-compatible supports. Typical operational conditions used in GAS produce NP beams that deposit on substrates with subsonic velocity [75]. If the range of masses of NPs is taken into account, one may conclude that NPs hit the substrate in a soft-landing regime in which the kinetic energy borne by a NP as a single entity is too small to break bonds between the highly numerous species constituting this NP. Hence, NP interaction with the substrate does not induce noticeable changes either in the NPs or in the substrates. Preservation of the NP shape and structure may or may not be of benefit, depending on the target application. A major drawback of the situation is related to the weak van der Waals forces acting between NPs and substrate so that a NP layer can be easily destroyed by a tiny mechanical impact. This drawback can be overcome by depositing a capping layer that should be sufficiently robust to fix the NPs on the surface yet sufficiently thin so as not to introduce morphological changes to the surface, unless otherwise required. The layer can be of the same or similar material as the underlying NPs to preserve the surface chemistry. For example, hydrocarbon NPs produced by plasma polymerization of *n*-hexane in GAS can be fixed on the surface by coating them with a thin film of hydrocarbon plasma polymer (Figure 14a). The capping layer can also be made of a different material as can be seen in Figure 14b where the same NPs are shown over-coated with a magnetron-sputtered Ti film.

**Figure 15:**
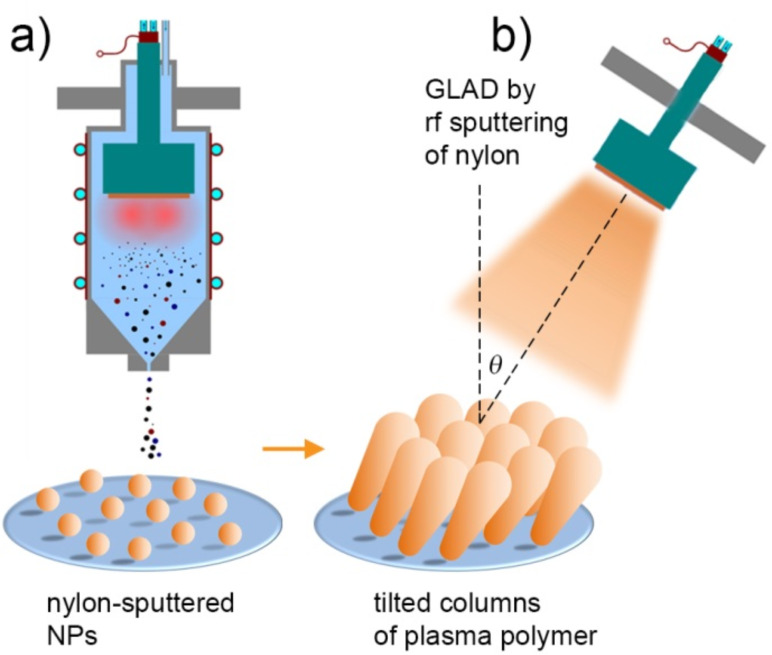
Scheme of GLAD: a) preseeding of substrates with nylon-sputtered NPs produced by GAS; b) GLAD of nylon-sputtered plasma polymer over the preseeded NPs.

In both of the above cases, the overlayer was deposited to preserve the initial surface morphology. Certain applications however may require control over the structure in a broader range covering both nanometer and micrometer scales. Plasma polymer NPs may be useful for this purpose as well, especially if glancing angle deposition (GLAD) is considered. Evaporative GLAD was developed in the late 20th century for creating metallic films with a highly porous structure [78]. It has been known that time and spatial fluctuations exist in atomic fluxes arriving onto the substrate from the gas phase during evaporation or sputtering. The noise in the atomic flux results in the situation that some spots on the surface may receive a larger amount of particles deposits than the others, and generally, noise is responsible for roughening the growing front. In contrast, surface diffusion tends to redistribute the arriving material over the larger area and leads to smoothening of the surface. The competition between the two phenomena determines the resultant roughness/smoothness of the film. If deposition is performed onto a sufficiently cold substrate, surface diffusion can be suppressed and roughening will dominate. It has been also recognized that roughening can be significantly enhanced if shadowing instabilities are present on the surface, especially if the depositing flux is collimated and tilted at an oblique (glancing) angle to the surface normal. Nuclei of the adsorbed material create shadow zones in areas opposite to the direction of the incoming flux. Arrays of well isolated zig-zag, spiral or pillar nanostructures made of metals and other inorganics have been successfully fabricated by GLAD. GLAD of polymeric materials has also been demonstrated [74,79–80]. However, the separation between the individual polymer nano-columns is typically worse than in the case of metals, probably due to a relatively large characteristic surface diffusion length of macromolecular species which compromises the shadowing effect. To enhance the shadowing mechanism, blank substrates can be preseeded with NPs produced by GAS (Figure 15a) that will serve as artificially created obstacles to the incoming flux in the second step (Figure 15b).

**Figure 16:**
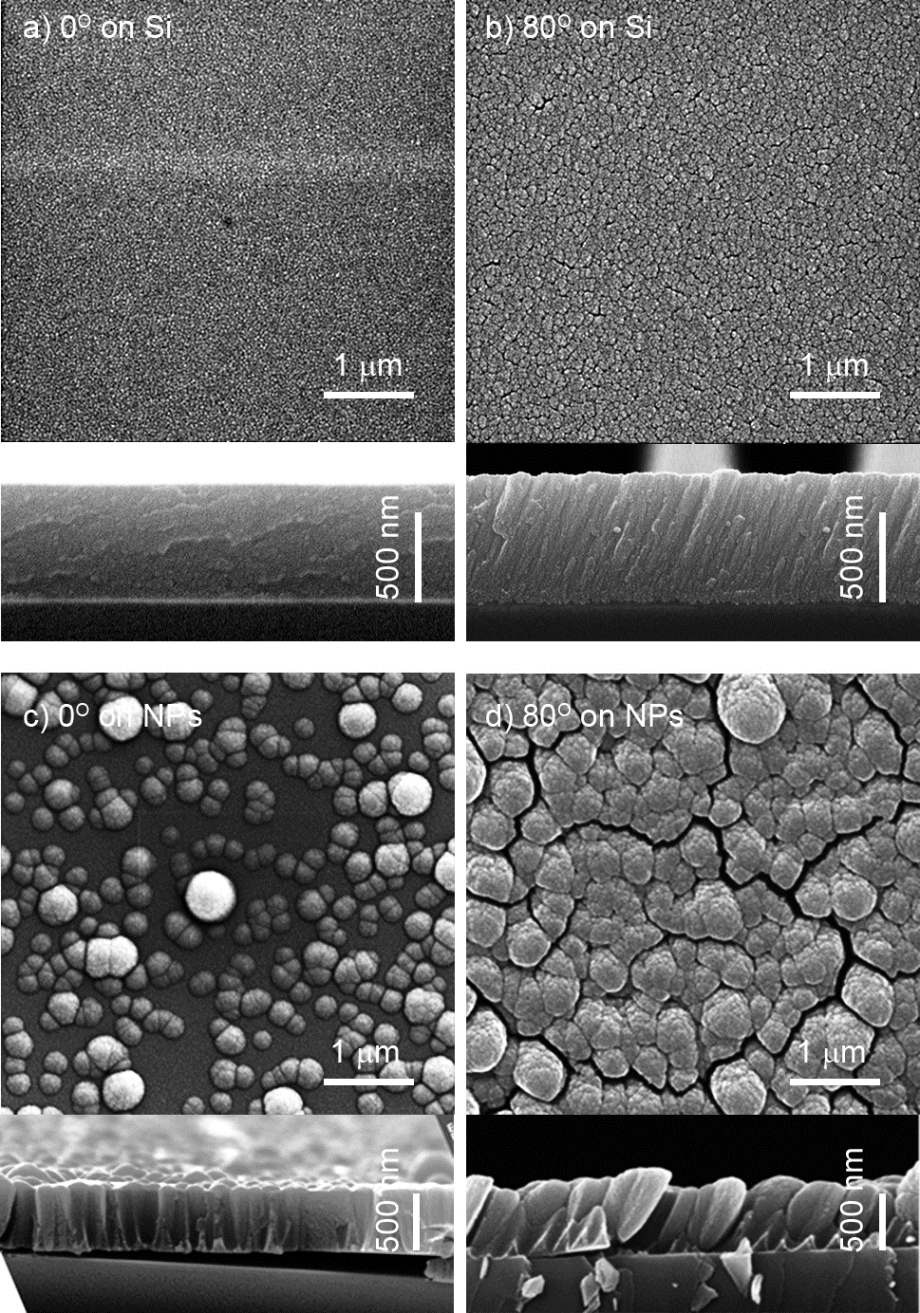
SEM images with combined top view and cross-sections of the deposits produced as a result of RF magnetron sputtering of nylon: a) normal deposition on blank Si substrate; b) GLAD at 80° on blank Si substrate; c) normal deposition over preseeded nylon-sputtered NPs; d) GLAD at 80° over preseeded nylon-sputtered NPs (reprinted from [81], with permission from Elsevier).

Figure 16a,b shows the top view and cross-sectional images of a plasma polymer film created as a result of RF magnetron sputtering of nylon at normal (0°) and glancing angle (80°) deposition on blank silicon substrates whereas Figure 16c,d shows their counterparts deposited over the preseeded nylon-sputtered NPs. As expected, normal depositions produce compact coatings with the surface replicating the underlying structure (correspondingly, smooth blank Si or roughened NP seeds). Using GLAD, a columnar structure develops with columns inclined towards the direction of the deposition. Obviously, the porosity of the coatings is greatly increased when it is deposited over the preseeded NPs. This approach also offers the possibility to combine different materials, and hence, to independently tune the surface morphology and the chemical composition. Thus, it represents an attractive route for designing nanocomposite coatings with advanced properties.

## Conclusion

Plasma polymer NPs have great potential and may provide a valuable addition to the field of nanoscale-dispersed polymers. The involvement of gas aggregation cluster sources in the production of plasma polymer NPs opens new horizons in precise tuning of their size, shape, chemical composition, surface charge and wettability. There are great potential benefits for the use of plasma polymer NPs in photonics, nanomedicine and other applications, but also significant challenges remain unresolved. Fundamental knowledge on the mechanisms of plasma polymer nanoparticle formation is still far from being complete. Although rich information regarding the nucleation and growth of nanoparticles in organosilicon and hydrocarbon plasmas is available, a deep understanding of these processes for other precursors is missing. The lack of knowledge is especially striking in the field of the plasma-based fabrication of functionalized polymeric NPs and core@shell NPs, which remains in its budding stage. The retention of specific functional groups, control over their concentration, control over the cross-link density and the concentration of radicals captured within plasma polymer NPs, control over the morphology and shape of NPs, and nanophase separation within core@shell NPs are only a few issues that scientists in this research field face. The solution of these issues requires finding correlations between the properties of the plasma (energy distribution functions, plasma density, floating and plasma potential), the gas phase composition and the gas flow dynamics. Therefore, future research work should join efforts of scientists with different expertise to cope effectively with these complex issues.
